# Development of a core outcome set for clinical trials in squamous cell carcinoma: study protocol for a systematic review of the literature and identification of a core outcome set using a Delphi survey

**DOI:** 10.1186/s13063-017-2069-2

**Published:** 2017-07-12

**Authors:** Daniel I. Schlessinger, Sanjana Iyengar, Arianna F. Yanes, Sarah G. Chiren, Victoria Godinez-Puig, Brian R. Chen, Anastasia O. Kurta, Jochen Schmitt, Stefanie Deckert, Karina C. Furlan, Emily Poon, Todd V. Cartee, Ian A. Maher, Murad Alam, Joseph F. Sobanko

**Affiliations:** 1 Department of Dermatology, Feinberg School of Medicine, Northwestern University, 676 North St. Clair Street, Suite 1600, Chicago, IL 60611 USA; 2 Department of Dermatology, St. Louis University School of Medicine, St. Louis, MO USA; 3 Centre for Evidence-Based Healthcare, Medizinische Fakultät Carl Gustav Carus, TU Dresden, Dresden, Germany; 4 Department of Dermatology, Penn State Health, Milton S. Hershey Medical Center, Hershey, PA USA; 5 Department of Otolaryngology, Feinberg School of Medicine, Northwestern University, Chicago, IL USA; 6 Department of Surgery, Feinberg School of Medicine, Northwestern University, Chicago, IL USA; 7 Department of Dermatology, Perelman Center for Advanced Medicine, Hospital of the University of Pennsylvania, Philadelphia, PA USA; 8 Division of Dermatologic Surgery, Department of Dermatology, Hospital of the University of Pennsylvania, Philadelphia, PA USA

**Keywords:** Core outcome set, Delphi, Consensus, Stakeholders, Squamous cell carcinoma, Systematic review

## Abstract

**Background:**

Squamous cell carcinoma (SCC) is a common skin cancer that poses a risk of metastasis. Clinical investigations into SCC treatment are common, but the outcomes reported are highly variable, omitted, or clinically irrelevant. The outcome heterogeneity and reporting bias of these studies leave clinicians unable to accurately compare studies. Core outcome sets (COSs) are an agreed minimum set of outcomes recommended to be measured and reported in all clinical trials of a given condition or disease. Although COSs are under development for several dermatologic conditions, work has yet to be done to identify core outcomes specific for SCC.

**Methods/design:**

Outcome extraction for COS generation will occur via four methods: (1) systematic literature review; (2) patient interviews; (3) other published sources; and (4) input from stakeholders in medicine, pharmacy, and other relevant industries. The list of outcomes will be revaluated by the Measuring PRiority Outcome Variables via Excellence in Dermatologic surgery (IMPROVED) Steering Committee. Delphi processes will be performed separately by expert clinicians and patients to condense the list of outcomes generated. A consensus meeting with relevant stakeholders will be conducted after the Delphi exercise to further select outcomes, taking into account participant scores. At the end of the meeting, members will vote and decide on a final recommended set of core outcomes. The Core Outcome Measures in Effectiveness Trials (COMET) organization and the Cochrane Skin Group - Core Outcome Set Initiative (CSG-COUSIN) will serve as advisers throughout the COS generation process.

**Discussion:**

Comparison of clinical trials via systematic reviews and meta-analyses is facilitated when investigators study outcomes that are relevant and similar. The aim of this project is to develop a COS to guide use for future clinical trials.

**Electronic supplementary material:**

The online version of this article (doi:10.1186/s13063-017-2069-2) contains supplementary material, which is available to authorized users.

## Background

Squamous cell carcinoma (SCC) is the second most common cutaneous malignancy. Although nodal metastasis of SCC is rare, “high-risk” tumors have an elevated 10–20% risk of metastasis [1–4]. High-risk SCC consists of lesions with size >2 cm, thickness/depth of invasion >4 mm, recurrent lesions, the presence of perineural invasion, a history of burn wounds or chronic inflammation, and immunosuppression. Treatments for SCC include standard surgical excision, Mohs micrographic surgery, cryotherapy, electrodesiccation and curettage, and radiation therapy [5].

Although authors of Cochrane reviews and other systematic reviews have considered the efficacy of various treatments for SCC, little research has been done to determine the most appropriate outcomes to assess those treatments [6]. Heterogeneity in outcomes measured across trials poses a concern when comparing the effects of different interventions. Selective outcome reporting bias, defined as results-based selection of outcomes for publication, is a problem in many clinical trials and affects the conclusions of a significant proportion of systematic reviews [7].

To address the inconsistencies present, organizations such as the Core Outcome Measures in Effectiveness Trials Initiative (COMET) bring together researchers interested in developing a standardized set of core outcomes in various health-related fields [8]. A core outcome set (COS) is defined as an agreed minimum set of outcomes that is recommended to be measured and reported in all clinical trials of a given condition or disease. Similarly, the Cochrane Skin Group - Core Outcome Set Initiative (CSG-COUSIN) was created to address COSs in dermatology by examining outcome measures in current research [9, 10]. CSG-COUSIN builds on the experiences of the Harmonizing Outcome Measures for Eczema (HOME) initiative, which developed a roadmap to guide the process of COS development and implementation [11–16]. Although COSs are under development for several dermatologic conditions, work has yet to be done to identify core outcomes specific for SCC. To minimize duplication, this study has been registered with the COMET and CSG-COUSIN organizations so that researchers are aware of our ongoing efforts and may participate if interested.

## Objective

The aim of this study is to develop an international COS relevant to clinical trials for the treatment of cutaneous SCC. Objectives include identifying the appropriate and relevant outcomes from all interventions and methods of assessment. The final core set of outcomes is recommended for inclusion but does not preclude other outcomes from being assessed.

## Methods/design

**Fig. 1:**
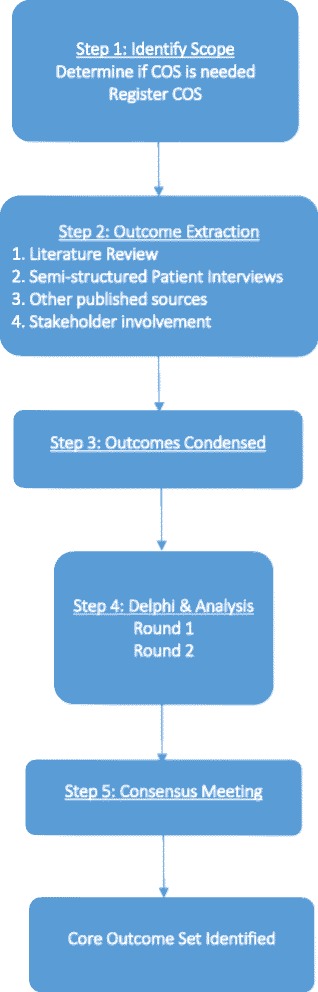
Flowchart of the study design. Modified from Schmitt et al. [16]. *COS* Core outcome set

The development of this COS adheres to the recommendations provided by the COMET and CSG-COUSIN initiatives, with reporting conforming to the Standard Protocol Items: Recommendations for Interventional Trials (SPIRIT) checklist (see Additional File 1) [8, 16]. This project has been adapted from a previously published protocol [17]. Figure 1 provides a brief overview of our study design.

### Scope of this COS

This COS is intended as the global/international standard for clinical trials examining the efficacy of various treatments for SCC. The COS may be applied to individuals of all ages, genders, races, and ethnicities. Similarly, both surgical and nonsurgical treatments may be evaluated using the outcomes generated from this study.

### Identification of outcomes

The list of outcomes currently reported will be generated over four phases:

1. *Phase I:* A systematic literature review will be performed to extract outcomes assessed in published randomized controlled trials and Cochrane reviews.
2. *Phase II:* Patient interviews will be conducted to determine patient-centered outcomes.
3. *Phase III:* Other sources, such as clinical trial registries and educational/treatment brochures, will be reviewed to ensure that all outcomes have been documented.
4. *Phase IV:* Additional stakeholders in medicine, pharmacy, and other relevant industries will be invited to provide insight into further outcomes that they would like included.

### Literature review

A systematic literature review using PubMed, Embase, and MEDLINE will be conducted, applying search terms related to randomized controlled studies for cutaneous SCC. Identical, published studies found across all the databases will be included once. Multiple publications on a single trial that report different outcomes or different follow-up times will be included as separate records. Authors, year of publication, source of funding, and intervention type will be documented among other study characteristics. Methodology, length of follow-up, treatment duration, results, outcomes, frequency of outcomes, and outcome measures will be noted. Outcomes extracted will then be placed into appropriate domains by two Measurement of Priority Outcome Variables in Dermatologic Surgery (IMPROVED) investigators. Similar outcomes will be listed only once. Combining and collapsing of outcomes will be performed judiciously to preserve content.

### Patient-centered outcomes

Patients will be recruited from among current patients of practicing physicians and skin cancer advocacy groups via emails and phone calls. Semistructured interviews will be conducted to explore domains identified in the literature review as well as other potential patient-identified outcomes. Open-ended questions will allow for patient expression of items important to them. Approximately 10–15 patients with SCC will be interviewed. A global context will be provided by including participants both in the United States and internationally. Interviews will be audio-recorded, transcribed, and coded to allow accurate and complete capturing of outcomes mentioned.

### Additional sources

Examination of other published sources, including clinical trial registries and Cochrane reviews, will be done to gather outcomes related to SCC. Pamphlets and brochures describing treatments and reported outcomes will be extracted with outcomes included in the final list.

### Stakeholder involvement

Stakeholders, or those invested in the development of a COS, will also be included in the decision process (Table 1). Dermatologists, drug and device safety regulators (e.g., U.S. Food and Drug Administration, European Medicines Agency), pharmacologists, pharmacists, and industry scientists associated with drugs and devices for treatment of SCC are potential members who can provide input regarding what outcomes they feel should be represented. Nurses, physician assistants, and other health care practitioners may be included as well to enhance further discussion.

**Table 1:** Summary of stakeholder involvement

Key stakeholders
Physicians (including dermatologists, international providers, physicians of other health care fields)
Patients
Drug and device safety regulators (e.g., FDA, EMA)
Pharmacologists/pharmacists
Industry scientists
Nurses, physician assistants, or other health care providers

*EMA* European Medicines Agency, *FDA* U.S. Food and Drug Administration

### Potential outcomes

The long list of outcomes obtained from the steps described above will then be examined by the steering committee, composed of four dermatologic surgeons: Drs. Murad Alam (Northwestern University), Ian A. Maher (St. Louis University), Joseph F. Sobanko (University of Pennsylvania), and Todd V. Cartee (Pennsylvania State University). Members may add or remove outcomes prior to the Delphi process. The steering committee members will not join in the Delphi process, but members will be invited to participate in the final consensus meeting.

### Delphi overview

Delphi surveys have been used in prior COS research [18]. The Delphi process involves a series of rounds of data collection and analysis to condense the opinions of individuals into a group consensus. Surveys can be conducted online through the use of specialized software. Responses to each round are collected, analyzed, and then redistributed to participants in successive rounds. We plan to conduct two Delphi rounds prior to the consensus meeting.

### Participants

Two separate, homogeneous groups composed of patients and physicians will participate in the Delphi exercises. Groups will consist of approximately 30 individuals to provide a diversity of input and account for potential dropouts. U.S. and international participants will be included. Prior to the exercise, details of the COS will be summarized and demographic/occupational information obtained, including years of experience, field of interest, and position. Consent will be assumed if participants complete the questionnaire. Participants will have 3 weeks to complete the online survey, with email reminders sent at the 1- and 2-week marks. For each round, the number of participants invited and those who complete the surveys will be documented.

### Delphi rounds

In the first Delphi round, the complete list of outcomes developed from the aforementioned steps will be presented for rating. Using a scale devised by the Grading of Recommendations Assessment, Development and Evaluation (GRADE) working group, participants will score each outcome on a scale from 1 to 9, with 9 being critically important and 1 being not that important [19]. For the first round, the additional option of 10 will be available if participants are unsure of the outcome’s need for inclusion. Participants will be asked to focus on ranking the most valued outcomes high and excluding outcomes of lesser importance. They will also have the option to add outcomes to the list that they feel should be included. All outcomes will be carried to the subsequent round.

Descriptive statistics will be used to analyze the data from the two groups. Responses from both stakeholder groups will be summarized and fed back to the participants after the first round, allowing participants to change their scores in light of others’ insights. Additionally, participants will be asked to identify new outcomes and determine if outcomes should be combined. New outcomes will be added to the list for the next round if two or more participants suggest its inclusion. Any uncertainties will be directed to the steering committee. The second Delphi round will follow the same format as the previous round. The set of outcomes resulting from this second round will be presented at the consensus meeting.

### Consensus meeting

Prior to solidifying a COS, a consensus meeting will be held to discuss all the results of the Delphi rounds and decide on the COS. Physicians, patients, and other stakeholders will be invited to the meeting to provide insight into the process. Results from each round of the Delphi survey will be presented. In terms of consensus, if 70% of participants rank the outcome 7, 8, or 9 with less than 15% scoring it 1–3, the outcome will be *retained* in the consensus pool [20]. Outcomes will be *removed* from the consensus list if 70% or more of the participants rank the outcome 1–3 and less than 15% rank the outcome 7, 8, or 9.

Feedback regarding the consensus-derived set of outcomes will then be elicited with the assistance of a trained moderator. Using live polling software, items will anonymously be voted for inclusion into the final core set of outcomes. If there are more than ten outcomes, then the steering committee will decide which outcomes will be kept through a discussion. By the end of the meeting, the goal is to create a COS of no more than ten outcomes that can be agreed upon by all stakeholders, patients, and physicians.

### Core outcome measures

Whereas the COS defines “what” to measure, the core outcome measurement instruments represent “how” to measure these domains. To define these measures, a systematic review of at least two databases will be done to identify currently used outcome instruments. The HOME roadmap will be used along with the COnsensus-based Standards for the selection of health Measurement INstruments (COSMIN) framework for guidance [16]. The quality of the studies will be assessed by rating their validity, reliability, responsiveness to change, and interpretability.

To determine which measurements are suitable per outcome domain, a consensus meeting with key stakeholders, patients, and clinicians will be held [16]. Results from the systematic review will be presented so that attendees can judge the measures on the basis of how valid, reliable, and feasible they may be as assessment tools. Each core outcome domain will be paired with a corresponding outcome measurement instrument. New instruments will be developed if there is inadequate evidence supporting existing methods. At the end of the consensus meeting, relevant stakeholders will vote to determine which measures should be included.

## Discussion

Use of heterogeneous or clinically irrelevant outcomes and outcome omission are increasingly problematic in the study of SCC treatment. The heterogeneity of these outcomes leaves clinicians unable to accurately compare findings in studies. Organizations such as COMET were formed to develop standardized core outcomes in various health-related fields. These COSs are not intended to limit the outcomes that can be measured but instead serve as a minimum of what should be measured. No COS for cutaneous SCC currently exists. The proposed protocol of COS generation for SCC will follow the COMET methodology with the aim of reducing the inconsistency of outcomes and outcome measurements across relevant trials.

### Trial status

The development of the COS is active and ongoing in its initial phase of outcome extraction.

## Additional file

Additional file 1: SPIRIT checklist. Completed checklist of the study protocol for the development of a core outcome set. (DOCX 48 kb)

## Abbreviations

COMET: Core Outcome Measures in Effectiveness Trials
COS: Core outcome set
COSMIN: COnsensus-based Standards for the selection of health Measurement INstruments
CSG-COUSIN: Cochrane Skin Group - Core Outcome Set Initiative
EMA: European Medicines Agency
FDA: U.S. Food and Drug Administration
GRADE: Grading of Recommendations Assessment, Development and Evaluation
HOME: Harmonizing Outcome Measures for Eczema
IMPROVED: Measurement of Priority Outcome Variables in Dermatologic Surgery
SPIRIT: Standard Protocol Items: Recommendations for Interventional Trials
